# Supplementary material for: First Case Reports of Nontuberculous Mycobacterial (NTM) Lung Disease in Ecuador: Important Lessons to Learn
Source: Pathogens. 2023 Mar 23;12(4):507. doi: 10.3390/pathogens12040507 (PMC10142742; doi:10.3390/pathogens12040507)
Supplement: Supplementary file 1 [file pathogens-12-00507-s001.zip › pathogens-2241618-supplementary.pdf]

pathogens

MDPI

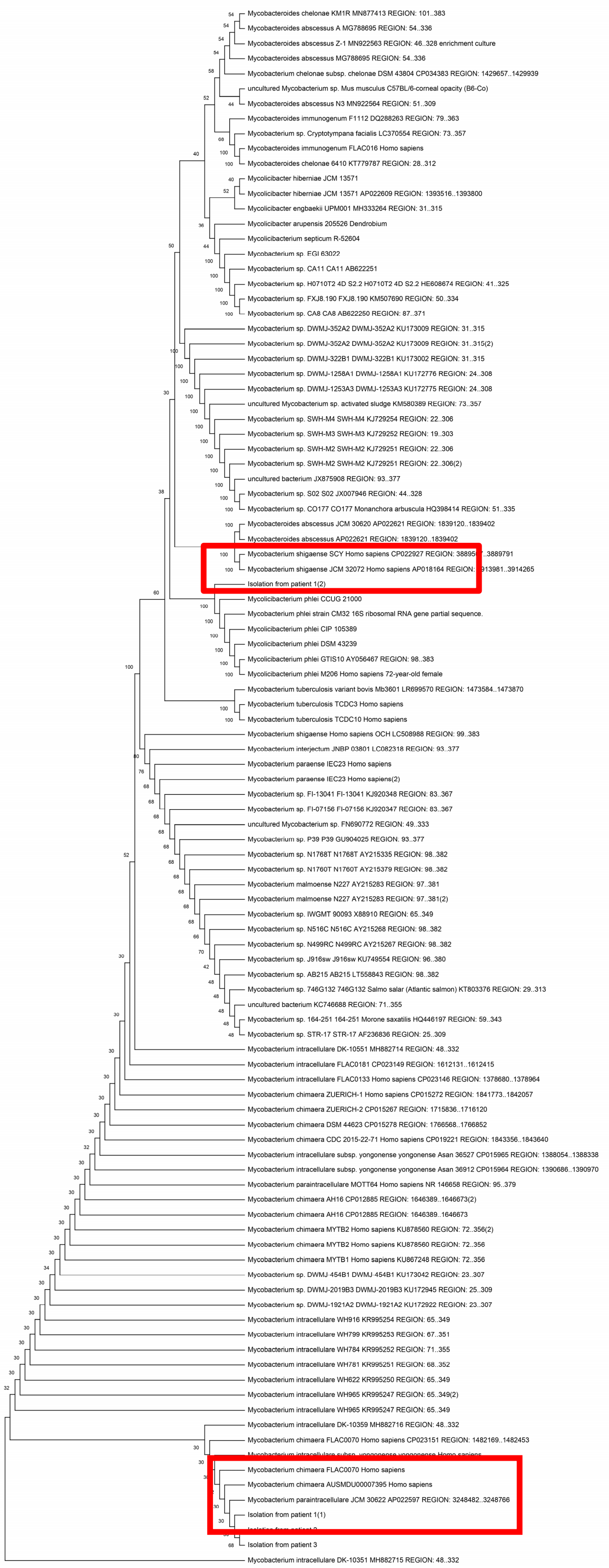

Supplementary Figure S1. A dendrogram of the 16S sequence from four NTM isolates recovered from a lung infection using the Neighbor-Joining algorithm in MEGA X software. The 16S rRNA tree, generated through a phylogenetic approach with sequences of reference strains, identified the

strains indicated in a red box. We used ML and T92+G+I with an MP guide tree to perform the phylogenetic construction, with the robustness of the bootstrapping set at 500 pseudo-replicates. See also reference 17.
